# Supplementary material for: Deletion of wfs1 Impairs Oligodendrocyte Precursor Cells Dorsal Distribution and Myelination Through the wfs1-hmgcs1 Axis in Zebrafish
Source: Biology (Basel). 2026 Aug 16;15(16):1402. doi: 10.3390/biology15161402 (PMC13510151; doi:10.3390/biology15161402)
Supplement: Supplementary file 1 [file biology-15-01402-s001.zip › File S1-Transcriptome sequencing data and differentially expressed genes (DEGs) in wfs1-- zebrafish at 5 dpf/2.KEGG/wfs1KOvsWT/UP/src/dre04623.html]

dre04623


- dre:562329

- Up regulated genes

562329(1.23033679822858)
- dre:394162

- Up regulated genes

394162\_1(1.13013950134542)

- dre:566185

- Up regulated genes

566185(6.72123660882883)

- dre:561021

- Up regulated genes

561021(2.14284154145004)

Close
